# Renal infarction in a patient with Coronavirus Disease 2019: another rare thrombotic event

**DOI:** 10.1590/0037-8682-0038-2021

**Published:** 2021-03-22

**Authors:** Luís Arthur Brasil Gadelha Farias, Eberson de Alcântara Cruz, Angélica Maria Holanda Pascoal da Silva, Tássia Ívila Freitas de Almeida

**Affiliations:** 1 Hospital São José de Doenças Infecciosas, Fortaleza, CE, Brasil.; 2 Hospital Geral de Fortaleza, Serviço de Clínica Médica, Fortaleza, CE, Brasil.

**FIGURE 1:**
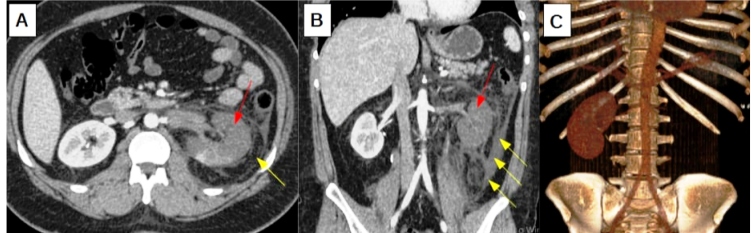
(A-C): Enhanced abdominal computed tomography. Left renal arterial thrombosis, with a marked reduction in renal perfusion (red arrows), with only a minimal uptake foci in the lower and middle third of this kidney. Important densification of the perirenal adipose planes with thickening of the anterior and posterior pararenal fascia as well as lateroconal fascia (yellow arrows).

A 37-year-old man presented to the emergency department with a 3-day history of nausea, vomiting, fever, and sudden low back pain in the left lumbar region that improved upon administration of 2 mg morphine every 4 h. Ten days before admission, he presented with cough, odynophagia, fever, and dyspnea, and tested positive for severe acute respiratory syndrome coronavirus 2 through nasopharyngeal swab test. Physical examination revealed a pain facies, no respiratory distress, respiratory rate of 20 rpm, heart rate of 118 bpm, and body temperature of 35.4 ºC. Abdominal examination revealed a positive Giordano sign in the left lumbar region. Normal vesicular breath sounds were recorded in both the lungs. Because of the history of sudden lumbar pain and fever, with high suspicion of vascular events or infectious complications, an enhanced abdominal computed tomography was performed, which revealed left renal arterial thrombosis with a marked reduction in renal perfusion, with only a minimal uptake foci in the lower and middle third of the kidney (Figure 1). After investigation, thrombophilia was ruled out. The transthoracic echocardiogram was normal. Blood culture samples were negative. Anticardiolipin immunoglobulin (Ig)M and IgG, β_2_-glycoprotein, and lupus anticoagulant tests were negative. Anticoagulant therapy was initiated with a 5-day regimen of 60 mg enoxaparin every 12 h followed by 5 mg/day warfarin without further adjustments.

Venous and arterial thrombosis and coagulation parameter abnormalities are well-known complications in the late phase of coronavirus disease 2019 (COVID-19)^1^. Few cases of renal infarction in COVID-19 patients have been reported^2^ ^,^ ^3^. It is hypothesized that the hypercoagulable state phenomena related to COVID-19 occur mainly through two mechanisms: disseminated intravascular coagulation and endotheliopathy, which generate thrombin, fibrin, and coagulation factors^3^. Further studies are necessary to understand the complete role of COVID-19 in vascular events.
